# A45 INFLIXIMAB IN COMBINATION WITH AN IMMUNOMODULATOR IS ASSOCIATED WITH AN ATTENUATED ANTIBODY RESPONSE TO BNT162B2 SARS-COV-2 VACCINE IN PEDIATRIC INFLAMMATORY BOWEL DISEASE PATIENTS

**DOI:** 10.1093/jcag/gwab049.044

**Published:** 2022-02-21

**Authors:** S Lawrence, Z Shire, F Reicherz, P Lavoie, K Jacobson

**Affiliations:** 1 Pediatric Gastroenterology, Hepatology and Nutrition, BC Children’s Hospital, Vancouver, BC, Canada; 2 BC Children’s Hospital, Vancouver, BC, Canada

## Abstract

**Background:**

Adult data have shown that Infliximab (IFX) impairs the antibody response to a single dose of the mRNA-BNT162b2 SARS-CoV-2 vaccine in patients with inflammatory bowel disease (IBD). The true impact of IFX on SARS-CoV-2 vaccine efficacy in pediatric IBD (PIBD) patients is unknown.

**Aims:**

To evaluate the humoral immune response to the BNT162b2 SARS-CoV-2 in PIBD patients treated with anti-tumor necrosis factor (TNF) therapy.

**Methods:**

PIBD patients treated with anti-TNF therapy either alone or in combination with an immunomodulator, who received at least one dose of the BNT162b2 SARS-CoV-2 vaccine, were prospectively enrolled from 1st June 2021 at BC Children’s Hospital. Serum antibody levels for [spike (S) protein and receptor-binding domain (RBD)] were determined at baseline and 28 days after their first and second vaccine doses. Antibody responses were assessed using multiplex serology IgG assay against four SARS-CoV-2 antigens: S-protein, RBD, N-terminal domain (NTD) and N-protein using the SARS-CoV-2 Panel 2 (Meso Scale Diagnostics).

**Results:**

Forty-two PIBD patients received a single dose of BNT162b2 (median age 14.5yrs (IQR 14–16); 43% female; 79% crohn’s disease, 21%, ulcerative colitis). Of those on IFX monotherapy (43%), both S-protein and RBD antibody concentrations 28 days post BNT162b2 were comparable to healthy adult controls (n=20, median age: 36yrs (IQR 29–40); 65% female) who had received one dose of BNT162b2 (p = 0.07) [Figure 1]. In PIBD patients on IFX in combination with either azathioprine or methotrexate (57%) both S-protein and RBD antibody concentrations were significantly lower than controls after 1 dose of BNT162b2 (p = 0.0003) [Figure 1].

In the PIBD cohort (n=27) who received 2 doses of BNT162b2 vaccine (median age 14yrs (IQR 14–16);41% female;63% crohn’s disease, 37% ulcerative colitis; median interval between doses 56 days (IQR 22–105)), there was no difference in antibody response after 2 doses compared to healthy adult controls (n=14, median age: 44 years (IQR 36–51); 29% female) whether they were on IFX monotherapy (41%) or in combination with an immunomodulator (59%) [Figure 1].

**Conclusions:**

We provide evidence of an attenuated antibody response in PIBD patients on IFX in combination with an immunomodulator after a single dose of BNT162b2. However, our data show a robust antibody response in PIBD patients, despite their infliximab treatment, after two doses of BNT162b2 vaccine. Our results are consistent with adult IBD data and highlight the importance of administering the second vaccine dose to achieve protection in this vulnerable patient population. Long-term follow-up to assess longevity of vaccine protection is warranted.

**Funding Agencies:**

None

**Figure:**
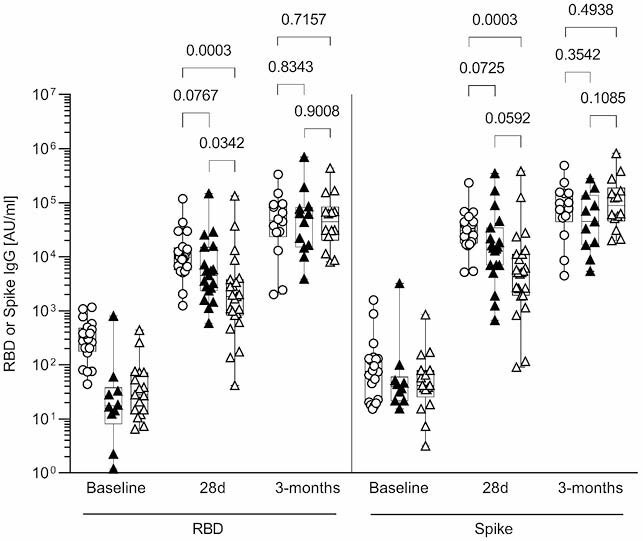
**Figure 1: SARS-CoV-2 IgG RBD and S-protein antibody levels [AU/ml] in PIBD patients and healthy adults before and after receiving the BNT162b2 SARS-CoV-2 vaccine.** IFX monotherapy (black triangles), IFX with immunomodulator (white triangles), healthy adults (white circles) at baseline, 28 days post first vaccine dose and 3-months post first vaccine dose (two doses of BNT162b2 vaccine). Data presented as boxes (IQR) and whiskers with p values

